# A critical review of the long-term disability outcomes following hip fracture

**DOI:** 10.1186/s12877-016-0332-0

**Published:** 2016-09-02

**Authors:** Suzanne M. Dyer, Maria Crotty, Nicola Fairhall, Jay Magaziner, Lauren A. Beaupre, Ian D. Cameron, Catherine Sherrington, for the Fragility Fracture Network (FFN) Rehabilitation Research Special Interest Group

**Affiliations:** 1 Department of Rehabilitation, Aged and Extended Care, School of Health Sciences, Flinders University, Adelaide, Australia; 2 Cognitive Decline Partnership Centre, The University of Sydney, Sydney, Australia; 3 The George Institute for Global Health, Sydney Medical School, The University of Sydney, Sydney, Australia; 4 Department of Epidemiology and Public Health, School of Medicine, University of Maryland, Baltimore, USA; 5 Departments of Physical Therapy and Surgery (Division of Orthopaedic Surgery), University of Alberta, Edmonton, Canada; 6 John Walsh Centre for Rehabilitation Research, Sydney Medical School Northern, The University of Sydney, St Leonards, Australia

**Keywords:** Hip fracture, Recovery of function, Mobility limitation, Activities of daily living, Institutionalisation, Quality of life, Osteoporosis, Aged, Longterm care, Review

## Abstract

**Background:**

Hip fractures are an increasingly common consequence of falls in older people that are associated with a high risk of death and reduced function. This review aims to quantify the impact of hip fracture on older people’s abilities and quality of life over the long term.

**Methods:**

Studies were identified through PubMed and Scopus searches and contact with experts. Cohort studies of hip fracture patients reporting outcomes 3 months post-fracture or longer were included for review. Outcomes of mobility, participation in domestic and community activities, health, accommodation or quality of life were categorised according to the World Health Organization’s International Classification of Functioning and synthesised narratively. Risk of bias was assessed according to four items from the Strengthening the Reporting of Observational Studies in Epidemiology (STROBE) statement.

**Results:**

Thirty-eight studies from 42 publications were included for review. Most followed a clearly defined sample from the time of fracture. Hip fracture survivors experienced significantly worse mobility, independence in function, health, quality of life and higher rates of institutionalisation than age matched controls. The bulk of recovery of walking ability and activities for daily living occurred within 6 months after fracture. Between 40 and 60 % of study participants recovered their pre-fracture level of mobility and ability to perform instrumental activities of daily living, while 40–70 % regained their level of independence for basic activities of daily living. For people independent in self-care pre-fracture, 20–60 % required assistance for various tasks 1 and 2 years after fracture. Fewer people living in residential care recovered their level of function than those living in the community. In Western nations, 10–20 % of hip fracture patients are institutionalised following fracture. Few studies reported impact on participation in domestic, community, social and civic life.

**Conclusions:**

Hip fracture has a substantial impact on older peoples’ medium- to longer-term abilities, function, quality of life and accommodation. These studies indicate the range of current outcomes rather than potential improvements with different interventional approaches. Future studies should measure impact on life participation and determine the proportion of people that regain their pre-fracture level of functioning to investigate strategies for improving these important outcomes.

**Electronic supplementary material:**

The online version of this article (doi:10.1186/s12877-016-0332-0) contains supplementary material, which is available to authorized users.

## Background

World-wide, people are living longer, with an increase in the proportion of older people in the population [1]. The greater understanding we have of the conditions that predominantly affect older people, the more prepared society can be to maximise health, opportunities and societal contributions for older people.

The health of bones, muscles and joints commonly deteriorates with advancing age. With increased age, there is a decrease in bone mineral density as well as muscle mass and strength [2] and the risk of falls and fall related injury increases [3]. The relative contribution of physiological ageing, chronic disease and inactivity to this deterioration is not yet understood but it is clear that there is an increased risk of fragility fractures, fractures that occur as a result of a low energy trauma (a fall from standing height or less) with increasing age.

Hip fractures are a common consequence of falls in older people and are particularly devastating in terms of their impact on an individual’s health and abilities. An estimated 95 % of hip fractures are due to falls [4]. The estimated annual prevalence of hip fractures globally is expected to reach 4.5 million by 2050 [5]. The highest aged-standardised rates of hip fracture due to osteoporosis are in North America and Europe [6]. Growth is also anticipated in other highly populated areas of the world, including countries in Asia, the Middle East and South America [1]. However while the age specific incidence is decreasing in a number of countries, population ageing in higher income countries is driving increases in the prevalence of hip fracture [7].

In high-income countries, most hip fractures are treated surgically, with admission to acute hospital care and, at times, a subsequent admission to a rehabilitation facility. Many older people who experience a hip fracture fail to fully recover. Many studies of hip fracture outcomes have been conducted but their findings regarding the long-term impact of hip fracture on independence in terms of mobility, activities of daily living (ADL) and life participation for older people are yet to be synthesised.

The aim of the current review was to provide an estimate of the impact of hip fracture on older people’s abilities over the long term. Large cohort studies reporting outcomes of activity and participation, health and quality of life 3 months or longer after hip fracture are reviewed.

## Methods

A critical review of cohort studies of hip fracture patients reporting outcomes of mobility, participation in domestic and community activities, health or quality of life at 3 months post-fracture or longer was conducted, following a protocol developed a priori. This review was developed within a rapid time frame and contains many similarities to evolving rapid review methodology [8]; it has a clearly defined review question based on the PICO (Population, Intervention, Comparator, Outcome) criteria, the review question and protocol were developed a priori, selection of studies is based on inclusion criteria and includes rigorous critical appraisal, summary and categorisation of the outcome data and interpretation of the findings is informed by the quality appraisal of the studies. Included studies had enrolled ≥75 % of patients aged over 60 years or the mean age was over 60 years, or enrolled only patients with a hip fracture due to a low energy trauma. Studies enrolling patients on the basis of the type of intervention received were excluded. Studies enrolling only those patients managed surgically (but ideally not a single type of surgery) were included as this is considered standard care in more developed countries.

Studies were identified by searching in PubMed, Scopus and hip fracture registries in November - December 2015 as well as through existing systematic reviews [9], reference lists of included studies and contact with experts.

Risk of bias was assessed according to four items adapted from the Strengthening the Reporting of Observational Studies in Epidemiology (STROBE) statement [10, 11] and Altman [12]; the items were rated as yes, no or unclear. The four items considered were (a) “Is it a representative sample?” this item was rated ‘yes’ if recruitment was consecutive or random; (b) “Were patients followed from inception?” This item was rated ‘yes’ if baseline time was same for all patients and close to fracture time, ratings considered whether or not patients were followed from inception rather than enrolled at inception– thus studies also scored ‘yes’ if data was recorded from time of admission; (c) “Is it a clearly defined sample?” This item was rated ‘yes’ if enrolment was for hip fracture according to hospital diagnosis with an age limit. Lastly, (d) “Was there adequate follow-up?” This item was rated ‘yes’ if more than 80 % of enrolled participants contributed follow-up data at 3 months or longer.

Results were extracted and outcomes categorised as primarily assessing activities (the execution of a task or action by an individual) or participation (involvement in a life situation) according to the World Health Organization (WHO) International Classification of Functioning, Disability and Health (ICF) framework [13]. Additional outcome categories were health condition, accommodations or quality of life.

It was not possible to synthesise all reported outcomes data from all studies identified that met the eligibility criteria. Thus this article includes and summarises data from the studies that reported outcomes as: (a) comparisons to a non-fracture group; (b) the time to recovery of function (or recovery at multiple follow-up times after hip fracture); (c) the proportion recovering pre-fracture function; (d) impact on function for those living in residential care. For outcomes where data was sparse, this was supplemented with some data from studies reporting outcomes at a single time point or from other countries. Where data were only reported in graphical form, they were not extracted for review. Outcomes at discharge or other time points earlier than 3 months were not extracted for review.

Proportion/percentage calculations from numerator and denominator were not verified, except where the data are presented in this review graphically. The proportion followed-up was calculated as the number of known deaths plus those with follow-up data as a proportion of those enrolled, wherever possible (ie known deaths + followed-up/ N enrolled). Where calculation of the proportion followed up could not be verified, the value as reported in the original publication was accepted. Where possible, the proportion of participants institutionalised (i.e., moving to residential aged care) was determined as the number institutionalised divided by the number surviving and not institutionalised at baseline, rather than the proportion of the total study population enrolled. The findings were synthesised in a narrative summary of the studies.

## Results

### Study characteristics

Data from 38 cohort studies reported in 42 publications were reviewed. Three studies (in four publications) reported outcomes in people sustaining a hip fracture within longitudinal population-based prospective cohort studies, thus pre-fracture ability and participation outcomes were recorded prospectively [14–17]. Eight studies reported outcomes for people following hip fracture in comparison to a non-fracture cohort [16–23]. In the remaining studies, pre-fracture function was determined retrospectively i.e. by participant or proxy interview after the fracture. Cohorts were primarily identified from hospital admission, surgery or discharge records or from government funding claims (eg Medicare in the USA).

A summary of the studies included, their size, country of origin and the outcomes included in this review is provided in Table 1. Seven studies were retrospective in design [12, 23–28], identifying hip fracture patients from hospital, health care or medical claims databases; the remainder were prospective (i.e. participants were identified on admission or discharge from hospital). Most studies stated that they excluded patients with pathological fractures, fractures secondary to other medical conditions or multiple fractures. One US study enrolled patients identified by Medicare claims into a longitudinal study with intentional oversampling of those 80 years of age or over, hence the average age was higher than in most other studies [14]. One study was a cohort of patients with trochanteric fractures treated by Ender nailing; this study was included as it reported on change in self-care outcomes, for which few studies were identified [29]. The majority of studies included patients aged 60 or 65 years and over, although nine studies included patients aged 50 years or over [12, 18, 24, 25, 30–36]. Eight studies specifically enrolled patients with hip fracture as a result of low-impact trauma or falls [12, 23, 27, 33, 36–39]; one included patients with fractures obtained from low to moderate energy trauma [30]. The remaining studies did not use the type of trauma that the injury was sustained from as an eligibility criterion. In most studies, three quarters or more of the participants were women. Three studies only enrolled women [12, 18, 40]. No studies reporting longer-term functional outcomes from hip fracture conducted in an African country were identified.

**Table 1:** Summary of included cohort studies, with outcomes included in review categorised using ICF framework^a^

Author, year	Country	N (hip fracture)	Recruitment	Activity			Participation			Health condition	Accomm	QOL	Mortality
				Mobility	Basic ADL	Self care	IADLs	Domestic	Community				
Relative to non-fracture group													
Autier 2000 [18]^b^	Belgium	170	1995–1996								Yes		Yes
Boonen 2004 [19]^b^	Belgium	170	1995–1996	Yes	Yes					Yes		Yes	Yes
Cumming 1996 [20]	Australia	131	1990–1992								Yes		Yes
Magaziner 2003^c^ [21]	USA	594	1990–1991	Yes		Yes							
Marottoli 1992^d, e^ [16]	USA	120	1982–1988	Yes		Yes							Yes
Norton 2000 [22]	New Zealand	911	1991–1994	Yes	Yes								
Tosteson 2001 [23]	USA	67	NR	Yes	Yes								
Wolinsky 1997^e^ [17]	USA	368	1984–1991	Yes	Yes		Yes	Yes					Yes
Population-based cohorts													
Bentler 2009 [14]	USA	495	1993–2005	Yes	Yes		Yes			Yes			Yes
Marottoli 1994^d^ [15]	USA	120	1982–1988								Yes		Yes
No comparison group													
Abimanyi-Ochom 2015 [30]	Australia	224	2009–2012									Yes	
Beaupre 2005 [50]^f^	Canada	919	1999–2000 & 1996–1997		Yes						Yes		Yes
Beaupre 2007 [48]^f^	Canada	451	1999–2000	Yes	Yes	Yes							Yes
Borgquist 1990 [31]	Sweden	103	1976–1977	Yes		Yes		Yes	Yes		Yes		Yes
Borgquist 1991 [32]	Sweden	837	1986–1988	Yes	Yes	Yes		Yes	Yes		Yes		Yes
Borgström 2013 [33]	International	1273	2003, 2007–2010									Yes	
Crotty 2000 [49]	Australia	215	1998–1999	Yes	Yes						Yes		Yes
Doshi 2014 [61]	Singapore	219	2011–NR		Yes								Yes
Givens 2008 [52]	USA	126	NR	Yes	Yes						Yes		Yes
Griffin 2015 [46]	UK	741	2012–2014	Yes								Yes	Yes
Holt 2008 [62]	Scotland	16380	1998–2005	Yes								Yes	Yes
Keene 1993 [41]	UK	1000	1989–1992	Yes				Yes				Yes	Yes
Kitamura 1998 [34]^g^	Japan	1169	1992	Yes	Yes	Yes					Yes		Yes
Tsuboi 2007 [35]^g^	Japan	963	1992	Yes							Yes		Yes
Koval 1998 [44]	USA	631	1987–1995	Yes									Yes
Koval 1998 [51]	USA	398	1988–1990		Yes		Yes						Yes
Magaziner 1990 [42]	USA	760	1984–1986	Yes	Yes		Yes						Yes
Magaziner 2000^c^ [43]	USA	674	1990–1991	Yes	Yes	Yes		Yes	Yes	Yes			Yes
Miller 2009 [40]	USA	205	1992–1995		Yes				Yes				
Morin 2012 [24]	Canada	12139	1986–2006								Yes		
Neuman 2014 [28]	USA	60111	2005–2007	Yes	Yes				Yes				Yes
Osnes 2004 [25]	Norway	1002	1996–1997	Yes	Yes						Yes		
Pereira 2010 [39]	Brazil	246	2001	Yes									Yes
Pitto 1994 [29]	Italy	143	1985–1987			Yes				Yes			Yes
Samuelsson 2009 [45]	Sweden	2134	2003	Yes	Yes						Yes		Yes
Shah 2001 [47]	USA	850	1987–1996	Yes	Yes		Yes				Yes		Yes
Ström 2008 [36]	Sweden	283	NR									Yes	
Suriyawongpaisal 2003 [26]	Thailand	250	NR	Yes		Yes						Yes	
Vergara 2014 [38]	Spain	638	NR		Yes		Yes						
Vochteloo 2013 [37]	Netherlands	390	2008–2009	Yes									Yes
Wang 2015 [12]	China	1151	2008–2012			Yes							Yes
Wong 2002 [27]	Singapore	274	1991–1993	Yes									Yes

^a^Yes indicates how the study outcomes are categorised according to the ICF framework

^b, c, d, f, g^ indicates hip fracture patients from same cohort

^e^Population-based cohort study with comparison to non-fracture group

*Accomm* accommodations, *ADL* activities of daily living, *IADL* instrumental activities of daily living, *ICF* International Classification of Functioning, *NR* not reported, *QOL* quality of life

### Risk of bias

Risk of bias assessment for the reviewed studies is shown in Table 2. Almost all included studies (37/42) followed participants from inception and included a clearly defined sample of participants with hip fracture. Two retrospective studies were enrolled via an invitation to provide follow-up data by questionnaire [23, 25]. The timing of enrolment was unclear in one Italian study [29] and no age limit for enrolment was reported in a large cohort from the United Kingdom (UK) and a retrospective study from Thailand (although all patients were over 50 years of age for the latter) [26, 41]. One fifth of the studies (8/42) did not include a representative sample and 36 % (15/42) of studies had inadequate follow-up, with follow-up data reported from less than 80 % of participants.

**Table 2:** Risk of bias assessment of included studies

Study	Representative^i^	Inception^j ^	Defined sample^k ^	Adequate Follow-up^l ^
Relative to non-fracture group				
Autier 2000^a^ [18]	Y	Y	Y	Y
Boonen 2004^a^ [19]	Y	Y	Y	Y^h ^(QOL = N)
Cumming 1996 [20]	Y	Y	Y	Y
Magaziner 2003^b ^ [21]	Y	Y	Y	Y
Marottoli 1992^c,d^ [16]	Y	Y	Y	Y
Norton 2000 [22]	Y	Y	Y	Y
Tosteson 2001 [23]	Y	N	Y	U
Wolinsky 1997^d ^ [17]	Y	Y	Y	N
Population-based cohorts				
Bentler 2009 [14]	N	Y	Y	N
Marottoli 1994^c^ [15]	Y	Y	Y	Y
No comparison group				
Abimanyi-Ochom 2015 [30]	U	Y	Y	N
Beaupre 2007 [48]^e^	Y	Y	Y	Y
Beaupre 2005 [50]^e^	Y	Y	Y	Y
Borgquist 1990 [31]	Y	Y	Y	Y
Borgquist 1991 [32]	Y	Y	Y	U
Borgström 2013 [33]	U	Y	Y	U
Crotty 2000 [49]	Y	Y	Y	Y
Doshi 2014 [61]	U	Y	Y	U
Givens 2008 [52]	Y	Y	Y	Y
Griffin 2015 [46]	Y	Y	Y	Y
Holt 2008 [62]	N	Y	Y	N
Keene 1993 [41]	Y	Y	N	Y
Kitamura 1998 [34]^f^	Y	Y	Y	U
Tsuboi 2007 [35]^f^	Y	Y	Y	U
Koval 1998 [44]^g^	N	Y	Y	N
Koval 1998 [51]^g^	Y	Y	Y	Y
Magaziner 1990 [42]	Y	Y	Y	Y
Magaziner 2000^b ^ [43]	Y	Y	Y	Y
Miller 2009 [40]	Y	Y	Y	N
Morin 2012 [24]	Y	Y	Y	U
Neuman 2014 [28]	Y	Y	Y	Y
Osnes 2004 [25]	Y	N	Y	Y
Pereira 2010 [39]	Y	Y	Y	Y
Pitto 1994 [29]	Y	U	Y	Y
Samuelsson 2009 [45]	Y	Y	Y	Y
Shah 2001 [47]	Y	Y	Y	Y
Ström 2008 [36]	U	Y	Y	Y
Suriyawongpaisal 2003 [26]	Y	N	U	U
Vergara 2014 [38]	U	Y	Y	Y
Vochteloo 2013 [37]	Y	Y	Y	Y
Wang 2015 [12]	Y	Y	Y	N
Wong 2002 [27]	Y	Y	Y	Y

^a, b, c, e, f, g^ indicates hip fracture patients from same cohort

^d^ Population-based cohort study with comparison to non-fracture group

^h^ For ADL and mobility outcomes

^i^ Rated yes if recruitment was consecutive or random; ^j ^rated yes if baseline time was same for all patients and close to fracture time; ^k ^rated yes if enrolment was for hip fracture according to hospital diagnosis with a defined age limit; ^l ^rated yes if ≥80 % of participants contributed follow-up data at ≥ 3 months

*QOL* Quality of Life, *Y* yes, *N* no, *U* unclear

### Activity outcomes

#### Mobility

Twenty-eight studies reported mobility outcomes (Table 1). Five studies provided a comparison to mobility outcomes for a non-fracture population (Table 3), four publications from three cohorts were population-based cohort studies [14–17] and 20 additional studies were cohorts with pre-fracture status determined retrospectively.

**Table 3:** Outcomes for hip fracture patients and control participants not experiencing hip fracture

Study	Outcome	Follow-up time	Controls matched for	Hip Fracture	Control	*P*-value
Activity - Mobility						
Boonen 2004 [19]	Unable to walk independently	1 year	age, residence			
	<80 years			30 %	7 %	<0.001
	>80 years			56 %	15 %	<0.001
Magaziner 2003 [21]	Disabled walking 3 m (SE)	1 year	age, gender, walking ability	54 % (2)	21 % (2)	<0.01
Marottoli 1992 [16]	Walk independently across room	6 mo (HF)	age, gender, physical function	15 %		NR
		1 year (Con)			72 %	
Norton 2000 [22]	Retain community mobility	2 years	age, gender	54 %	87 %	*P* < 0.001^e^
Wolinsky 1997 [17]	Mean increase in no. lower body limitations	Median 2.3 years	nil^f^	1.75	0.75	*P* ≤ 0.0001
	Mean increase in no. upper body limitations			0.50	0.27	*P* < 0.001
Activity - Composite measure of Basic ADLs						
Boonen 2004 [19]	Mean RDRS-2 score for assistance with ADL (95 % CI)	1 year	age, residence	8.6 (7.5–9.9)	2.8 (2.1–3.4)	<0.001
Norton 2000 [22]	Retain functional independence	2 years	age, gender, independence	72 %	94 %	*P* < 0.001^e^
Tosteson 2001 [23]	Limited daily activities	1–5 years	nil	59 %	13 %	<0.05^c^
Wolinsky 1997 [17]	Mean increase in no. ADL limitations	Median 2.3 years	nil^f^	2.08	0.79	*P* ≤ 0.0001
Activity - Self-care						
Magaziner 2003 [21]	Requiring assistance with grooming (SE)^i ^	1 year	age, gender, walking ability	17 % (2)	9 % (1)	*P* < 0.001
		2 years		18 % (2)	10 % (1)	*P* < 0.001
Marottoli 1992 [16]	Dressing independently	6 mo (HF)	age, gender, physical function	49 %	-	NR
		1 year (Con)		-	91 %	
Tosteson 2001 [23]	Difficulty putting on socks	1–5 years	nil	43 %	13 %	*P* < 0.05
Participation – domestic life						
Wolinsky 1997 [17]	Mean increase in no. household ADL limitations^g^	Median 2.3 years	nil^f^	0.89	0.45	*P* ≤ 0.0001
Participation – IADLs						
Wolinsky 1997 [17]	Mean increase in no. advanced ADL limitations^h ^	Median 2.3 years	nil^f^	0.44	0.26	*P* < 0.01
Health condition						
Boonen 2004 [19]	Mean RDRS-2 score (95 % CI):	1 year	age, residence			
	Dependence^b^			3.1 (2.6–2.7)	1.0 (0.7–1.3)	<0.001
	Cognitive impairment			0.9 (0.7–1.1)	0.3 (0.2–0.4)	<0.001
Accommodation						
Autier 2000 [18]	Institutionalisation	1 year	age, residence	20 %	4 %	
Cumming 1996 [20]	Institutionalisation	1 year	nil	27 %	5 %	<0.05^d^
Quality of life						
Boonen 2004 [19]	Mean (95 % CI) RDRS-2 score for QOL (inverted, higher indicates poorer QOL)	1 year	age, residence	38.9 (34.3–43.5)	31.5 (27.5–37.5)	<0.001
Tosteson 2001 [23]	Mean QALY (95 % CI)	1–5 years	nil	0.63 (0.52, 0.74)	0.91 (0.88, 0.94)	<0.051^a^

*Abbreviations: ADL* activities of daily living, *Con* control, *HF* hip fracture, *mo* months, *NR* not reported, *QALY* quality adjusted life years, *QOL* quality of life, *RDRS-2* Rapid Disability Rating Scale version-2, *SE* standard error

^a^Difference remained after adjustment for age and hormone replacement therapy use

^b^For hearing, sight, communication, staying in bed during the day, incontinence and medication

^c^Difference remained after adjustment for age

^d^HR significantly different to 1.0 (HR = 4.0, 95 % CI 1.7 – 9.5) after adjustment for age, sex, mental state score, use of proxy respondent, living alone, living with spouse, physical activity (time spent working and/ or walking), number of self-reported medical conditions and self-reported history of myocardial infarction or Parkinson’s disease

^e^After controlling for differences in age, gender and baseline mobility/functional independence

^f^Controls represent those in the prospective cohort that did not experience hip fracture

^g^Includes four items from Duke: meal preparation, shopping, light and heavy housework

^h^Includes managing money, using telephone and eating

^i^Control cohort reported is Iowa EPESE cohort; two other control cohorts also reported, with consistent findings

##### Comparison to a non-fracture group

Studies from three different countries demonstrated that mobility 1 to 2 years following hip fracture is significantly worse than for matched control subjects (Table 3) [19, 21, 22]. A United States (US) study of people residing in the community estimated that the excess number of people disabled after 2 years was 26 per 100 people with hip fracture for walking 3 metres (10 ft) and 22 per 100 for bed transfers [21]. A New Zealand study reported that people experiencing hip fracture were four times more likely to be unable to mobilise in the community 2 years after fracture (OR 4.2, 95 % CI 2.8–6.2, *p* < 0.001) [22].

A US population-based cohort study of ageing found those sustaining a hip fracture had a statistically significant increase in the number of upper and lower body limitations 2 years after the fracture, in comparison to the remainder of the cohort [17]. The mean increase in the number of limitations in those with hip fracture was 0.93 for the lower body (*p* = 0.0001) and 0.26 for the upper body (*p* = 0.02), in comparison to those without hip fracture, after adjusting for baseline differences between the groups [17]. However, there is some uncertainty in these findings as fewer than 80 % of hip fracture patients contributed follow-up data in this study.

##### Time to recovery

Two US studies from the same US centre provided estimates of the time to recovery (Additional file 1: Table S4) [42, 43]. A study of hip fracture patients from the 1980s found that most patients who recover their pre-fracture walking ability do so within the first 6 months after discharge from hospital [42]. However, over the following 6-month period, a further 10 % of patients regained their walking ability while a similar proportion declined. Time for recovery of different mobility activities varied, ranging from approximately 10 months for chair rise speed to 14 months for walking 3 metres (10 feet) without assistance [43].

Two studies have indicated that the proportion recovering their pre-fracture mobility can continue to increase beyond 3 months (Table 4) [37, 44]. However as these data represent mobility in survivors, this may indicate longer survival of those with better mobility. Seven studies reported absolute walking or mobility rates at multiple time points following hip fracture (see Additional file 1: Table S1). In Swedish and Japanese studies, the proportion of surviving patients walking was found to be similar at 4 months and 1 to 2 years [31, 34, 45] and in community living people the proportion of survivors walking remained relatively constant to 10 years [31]. In contrast, a UK study found some increase in the proportion walking at 1 year [46].

**Table 4:** Proportion of survivors that recover their pre-hip fracture levels of activity, participation or health outcomes

Study	Outcome measure	Pre-fracture residence	Surgical cohort	3–4 months	6 months	1 year	2 years
Activity – Mobility							
Bentler 2009 [14]	Mobility activities without difficulty^e^	NR	N				47 %
Crotty 2000 [49]	Level of ambulation^b^	Community	Y	69 %			
		LTC	Y	58 %			
Holt 2008 [62]	Walk unaided and unaccompanied	Mixed	Y				
	Ages 75–89			22 %			
	Ages ≥95			2 %			
Keene 1993 [41]	Walk unaided	Mixed	N			40 %	
Koval 1998 [44]^g^	Ambulatory ability	Community	Y	22 %	38 %	47 %	
Shah 2001 [47]^g^	Ambulation independence	Community	Y			44 %	
Magaziner 2000 [43]	Walk 3 m without assistance^a, d^	Community	N			60 %	63 %
Norton 2000 [22]	Retain community mobility^d^	Mixed	U				54 %
Osnes 2004[25]	Walking independence^f^	Mixed	U			44 %	
Pereira 2010 [39]	Remain stable on BOAS^d^					55 %	
Vochteloo 2013 [37]	Mobility	Mixed	Y	46 %		48 %	
	Mobility without aid		Y	27 %		40 %	
	Mobility with aid		Y	58 %		58 %	
Activity – Composite measure of Basic ADLs							
Bentler 2009 [14]	ADLs without difficulty^e^	NR	N				49 %
Beaupre 2005 [50]^h^	ADL level (MBI)	Mixed	Y	34 %	42 %		
Beaupre 2007 [48]^h^	ADL level (MBI)	Community	Y		71 %		
		LTC	Y		22 %		
Givens 2008 [52]	ADL no decline^b, c^	Mixed	Y		71 %		
Koval 1998 [51]^g^	ADL level	Community	Y	59 %	71 %	73 %	
Shah 2001 [47]^g^	ADL level	Community	Y			70 %	
Norton 2000 [22]	Functional independence^d^	Mixed	U				72 %
Osnes 2004 [25]^f^	Living at home receiving assistance, assistance received at same frequency	Mixed	U			49 %	
	Living at home without assistance					45 %	
Vergara 2014 [38]	ADL (MBI)^b^	Mixed	U		29 %		
Activity – Self-care							
Magaziner 2000 [43]^a, d^	Washing	Community	N			62 %	56 %
	Dressing (socks & shoes)					67 %	67 %
	Dressing (pants)					80 %	80 %
	Getting on/off toilet					36 %	37 %
Activity – Communications							
Magaziner 2000 [43]	Using the telephone^a, d^	Community	N			78 %	77 %
Participation – Composite measures of Instrumental activities of daily living (IADLs)							
Bentler 2009 [14]	IADLs without difficulty^e^	NR	N				55 %
Koval 1998 [51]^g^	IADLs	Community	Y	34 %	42 %	48 %	
Shah 2001 [47]^g^						46 %	
Vergara 2014 [38]	IADLs^b^	Mixed	U		25 %		
Participation – Domestic life							
Magaziner 2000^a, d^ [43]	Housecleaning	Community	N			38 %	57 %
	Shopping					58 %	59 %
	Cooking					76 %	77 %
	Handling money					69 %	69 %
Pitto 1994 [29]	Social function (mix of self and domestic care)^b, d^	Mixed			60 %		
Participation – Community, social and civic life							
Magaziner 2000 [43]	Getting places out of walking distance^a, d^	Community	Y			47 %	47 %
Health condition							
Bentler 2009 [14]	Self-reported health status^b^	NR	N				61 %
	Cognition (TICS)^b^						56 %
Magaziner 2000 [43]	Taking medications^a, d^	Community	Y				71 %
Pitto 1994 [29]	Health status^b, d^	Mixed		64 %			82 %

^a^Determined as 100 % less percentage of survivors newly dependent

^b^Determined as 100 % less the percentage of survivors deteriorated

^c^In this study, for patients who had died, functional status in the 2 weeks before death was determined by proxy interview and included

^d^n/N not confirmed

^e^Determined as 100 % less the percentage of survivors that got worse regarding the number of activities with difficulty

^f ^Determined as 100 % less the percentage with loss of walking independence/receiving assistance, participants not followed from inception

^g, h^Studies from the same cohort

*ADL* activities of daily living, *BOAS* Brazil Old Age Schedule, *LTC* long term care, *MBI* modified Barthel Index, *N* no, *NR* not reported, *TICS* Telephone Interview to Assess Cognitive Status, *U* unclear, *Y* yes

##### Recovery of pre-fracture function

Data from 10 cohort studies reporting the proportion of people experiencing hip fracture that regain their level of mobility are summarised in Table 4. In general, data from the included cohort studies indicate that 40 to 60 % of surviving patients regain their pre-fracture level of mobility within 1 year (Table 4) [25, 37, 39, 41, 43, 44, 47]. Studies from the US, United Kingdom (UK) and the Netherlands found that amongst those who were independently mobile pre-fracture, 40–44 % of patients recover their pre-fracture mobility independence [25, 37, 41, 47].

Two US population-based studies recorded pre-fracture mobility prospectively (see Additional file 1: Table S2). One found that 53 % of patients had worse mobility 2 years following hip fracture [14]. In this study, the population source had oversampled people 80 years or older and those included were self-respondents at the interview immediately before and after hip fracture. Those that were excluded because of proxy respondents had the worst baseline function. Also, unfortunately, fewer than 80 % of subjects contributed follow-up data in this study. The other study reported that the proportion of survivors able to walk across a room independently decreased from 75 % pre-fracture to 15 % at 6 months [16]. Follow-up for this study was considered adequate, with more than 80 % contributing data.

##### Outcomes for people living in residential care pre-fracture

Most studies were conducted in populations from the general community or from settings with a minority of people from residential care. Four articles on three cohorts reported functional outcomes for people in residential care [28, 48–50]. Canadian and Australian studies have found that recovery of mobility is lower for those living in residential care than for those living in the community (Table 4; Additional file 1: Table S3) [48, 49]. A large retrospective study of people living in nursing homes in the US reported that, of those who were independently mobile pre-fracture, only 21 % survive and regain their pre-fracture independence at a median of 4 months [28]. This outcome measure differs to those presented in Table 4 for other studies, as the rate indicates a composite of recovery and survival, rather than representing the rate of recovery as a proportion of those surviving. Of those with follow-up data and surviving one year after fracture, 27 % had new total dependence in locomotion (Additional file 1: Table S3) [28].

It should be noted that the pre-fracture mobility of the people in these studies was determined retrospectively, so there is a risk of bias in these data.

#### Composite measures of basic activities of daily living

##### Comparisons to a non-fracture group

A Belgian study comparing hip fracture patients to matched controls estimated that 24 % of the loss of independence in activities of daily living (ADLs) 1 year after hip fracture is directly attributable to hip fracture (Table 3) [19]. Other studies indicate that survivors are significantly more likely to be functionally dependent 2 years after fracture (adjusted OR 2.6, 95 % CI 1.7–4.1, *p* < 0.001) [22] and have more difficulties with activities of daily living [17, 23].

##### Time to recovery

Two US studies from the same US centre provided estimates of the time to recovery (Additional file 1: Table S4) [42, 43]. Most patients who recover their ability to perform basic ADLs did so within the first 6 months after discharge from hospital, but over the following 6-month period a similar proportion improved or declined (Additional file 1: Table S4) [42]. Time for recovery for different activities ranged from approximately 4 months for upper extremity activities of daily living to 11 months for lower extremity physical ADLs (Additional file 1: Table S4) [43].

Two studies found the proportion of patients recovering their pre-fracture level of ADL increased between 3 and 6 months (Table 4) [50, 51]. However, as these data represent function in survivors, this may indicate longer survival of those better functioning.

##### Recovery of pre-fracture function

Data from seven cohort studies reporting the proportion of survivors to recover their pre-fracture level of independence for ADLs are summarised in Table 4. Separate studies conducted in the US and Canada estimated that 34 to 59 % of patients regain their pre-level of fracture basic ADL function by 3 months [50, 51] The proportion rises to 42 to 71 % by 6 months [50, 51]. Three separate studies have indicated that approximately 70 % of survivors can recover their pre-fracture level of ability for basic ADLs [22, 47, 51, 52].

Two US population based cohorts found a significant increase in the number of basic ADL limitations for those who had experienced hip fracture compared to older people who had not [17]. Another US population based study found that 51 % of people had deteriorated in terms of basic ADLs after 2 years (Table 3, Additional file 1: Table S2), however follow-up was considered inadequate in these studies [14].

Data from studies reporting composite basic ADL outcomes at multiple time points following hip fracture generally demonstrate that while approximately 60 to 75 % of people are independent in basic ADLs pre-fracture, this decreases to 40 to 60 % post-fracture (Additional file 1: Table S1). A Norwegian study reported that half of all patients who were living at home before and after hip-fracture, but not receiving assistance pre-fracture, were receiving assistance 1 year after fracture [25]. For those living at home with assistance before and after fracture, half received assistance at the same frequency after fracture (Table 4) [25]. This study did not follow participants from recruitment but surveyed survivors retrospectively using a questionnaire, there is a high risk of bias in these data as a greater proportion of non-responders were discharged to nursing homes.

##### Outcomes for people living in residential care pre-fracture

Two studies from Canada and Australia indicate that those living in long-term care before a fracture have a poorer recovery than those living in the community (Table 4, Additional file 1: Table S3) [48, 49]. At 6 months post-fracture, a greater proportion of those living in the community before the fracture recovered their pre-fracture function, in comparison to those living in long term care (Table 4*p* < 0.001) [48]. The adjusted reduction in pre-fracture function at 6 months was 33 % (95 % CI −40.6 to −27.2) for those in long-term care, and 12 % (95 % CI −14.8 to −8.4) for those community dwelling before the fracture [48]. It should be noted that the pre-fracture mobility of the people in all of these studies was determined retrospectively, so there is a risk of bias in these data.

#### Self-care

##### Comparison to a non-fracture group

Three studies have reported that fewer people who sustained a hip fracture were independent in self-care outcomes of dressing and grooming in comparison to a non-fracture cohort (Table 3).[16, 21, 23] Magaziner et al. [21] estimated that 2 years following hip fracture, six additional people are disabled in personal grooming, per 100 hip fractures, in comparison to a non-fracture control group.

A US study found a significantly greater proportion of people experiencing hip fracture had difficultly putting on socks 1 to 5 years following hip fracture, in comparison to those without a fracture (Table 3) [23]. However, there is uncertainty in these findings, as the control participants were not matched, the participants were not followed from inception and it is unclear whether follow-up was adequate.

##### Recovery of pre-fracture function

Magaziner et al. [43] found that of patients who were independent in performing various self-care activities (including washing and dressing) before hip fracture, approximately 20 to 65 % require assistance to do these tasks 1 and 2 years following the fracture (Table 4). In a small Italian study, 40 % experienced deterioration in a measure of level of dependence in both personal and domestic needs 6 months post-fracture (Table 4) [29].

One population-based study that recorded pre-fracture ability prospectively found that while 86 % of people could dress independently before the fracture, only 49 % were independent with dressing at 6 months (Additional file 1: Table S2) [15]. A similar decrease in independence was seen in the subgroup of hip fracture patients that could perform four of five activities independently before the fracture (99 % independent dressing at baseline versus 60 % at 6 months, Additional file 1: Table S2).

Cohorts from Sweden, Japan, Canada and the US in which all or most people were living in the community indicated that 70 % or more of hip fracture survivors were independent in dressing outcomes beyond 4 months post-fracture [31, 34, 48], with rates staying similar up to 10 years post-fracture in a small community living cohort (Additional file 1: Table S1) [31]. This was consistent with outcomes from a Thai study population with a high degree of independence pre-fracture (Additional file 1: Table S5) [26]. A large, retrospective cohort study of data from 18 hospitals in China reported that approximately 60 % of women experiencing a fracture from low impact trauma were independent in self-care a median of 2.6 years after the fracture (Additional file 1: Table S5). Data on the level of independence before the fracture were not available [12].

##### Outcomes for people living in residential care pre-fracture

A Canadian study conducted in patients from mixed settings reported that approximately 60 % of patients overall were independent with dressing at 6 months post-fracture, compared with 74 % pre-fracture [48]. This represented 73 % independent for those living in the community before the fracture, and 9 % for those living in long-term care; 75 % of patients in the study were from the community (Additional file 1: Table S3) [48].

### Participation outcomes

#### Composite measures of instrumental activities of daily living

##### Comparison to a non-fracture group

In the US Longitudinal study of Ageing, there was a statistically significant increase in the mean number of advanced ADL limitations in hip fracture patients over the first two postoperative years, in comparison to older adults who did not experience a hip fracture (Table 3) [17].

##### Time to recovery

A study of hip fracture patients from the US in the 1980s found that most patients who recover their ability to perform IADL do so within the first 6 months [42]. The proportion who were fully independent was greater at 6 months post-discharge than at 2 months post-discharge (Additional file 1: Table S1) [42]. Over the following 6-month period, approximately 20 % of patients improved further while a similar proportion declined to the same degree (Additional file 1: Table S4) [42].

##### Recovery of pre-fracture function

Two US studies indicated that approximately half of all patients have recovered their pre-fracture independence in terms of IADL 1 or 2 years following fracture (Table 4) [14, 47]. In a US community living cohort, 34 % of people were fully independent in IADL at pre-fracture baseline; this declined to 14 % after 1 year [42]. However Spanish data indicated that only 25 % had recovered at 6 months post-fracture (75 % had deteriorated, deterioration was defined as a score of less than 5 points or a decrease of 2 points on the Lawton IADL scale) [38]. Pre-fracture ability was determined retrospectively in these studies, so there is a risk of bias in these data.

#### Domestic life

##### Comparison to a non-fracture group

One study provided data on domestic participation for people experiencing hip fracture in comparison to a non-fracture group. The Longitudinal Study of Ageing reported a statistically significant increase in the number of household ADL limitations experienced by older Americans who had had a hip fracture, in comparison to the remainder of the cohort (Table 3) [17].

##### Time to recovery

Two studies reported participation in domestic life at more than one time point following hip fracture [31, 43]. A small Swedish study of community living people found that while the proportion of people who participated in domestic life was reduced 4 months following hip fracture; participation remained at approximately the same level for those alive 10 years after their fracture (Additional file 1: Table S1) [31]. Approximately one third of people had the need for social services after fracture; this proportion remained similar from 4 months to 10 years after fracture (Additional file 1: Table S1) [31]. The US study reported similar rates of dependence 1 and 2 years post-fracture [43].

##### Recovery of pre-fracture function

Magaziner et al. [43] reported that in a cohort of patients living in the community pre-fracture, approximately 20 to 60 % of patients were dependent on others for performance of various domestic activities 1 year after fracture (Additional file 1: Table S1). In a large UK cohort, participation in shopping decreased from 54 % pre-fracture to 33 % of survivors after 1 year [41].

#### Community, social and civic life

##### Recovery of pre-fracture function

Four studies from two centres reported data on participation in community or social life after hip fracture [31, 32, 40, 43]. No studies were identified reporting on participation in civic life (eg. voting). A US cohort found that 1 and 2 years after hip fracture, 53 % of those independent pre-fracture required assistance to access, or could not get to, places out of walking distance (Table 4) [43].

In terms of social participation, another cohort from the same region (Baltimore, USA) found that on average, social participation after 6 months for those who fell once or less was similar to that before their fracture, but was reduced in those experiencing two or more falls (Additional file 1: Table S1) [40]. Swedish studies found that for hip fracture patients who were living in their own home pre-fracture, the proportion of people that visited someone in the last month was similar both before, 4 months, 5 and 10 years after fracture (Additional file 1: Tables S1 and S5) [32].

#### Health condition

##### Comparison to a non-fracture group

A Belgian study reported that the degree of dependence and of cognitive impairment was significantly worse for women 1 year after hip fracture than for women who had not experienced fracture (Table 3) [19].

##### Time to recovery

Magaziner et al. [43] reported that the recovery time for cognitive impairment following the hospital stay was 4.4 months.

##### Recovery of pre-fracture health status

Three cohort studies reported medium- to long-term health condition outcomes following hip fracture. A US population-based longitudinal study recording health status prospectively before fracture reported that 2 years following fracture nearly half of patients experiencing hip fracture reported a decline in their health status or cognition (Table 4) [14]. It should be noted that the population source for this study had oversampling of people 80 years or older [14].

Magaziner et al. [29] reported that 1 and 2 years after hip fracture, almost 30 % of those taking medications independently pre-fracture required assistance to do so (Table 4) [43]. A small Italian cohort of patients treated with Ender nailing found that approximately one third of patients reported a deterioration in their health status 6 months after fracture; 5 years after fracture this was approximately one fifth (Additional file 1: Table S1).

#### Accommodation

##### Comparison to a non-fracture group

Two studies from different countries have reported an increased risk of institutionalisation in the year post-fracture for people experiencing hip fracture in comparison to controls (Table 3; Belgian study RR for survivors 5.6, 95 % CI 2.0–15.6 [18]; Australian study HR 4.0, 95 % CI 1.7–9.5 risk adjusted for multiple health-related factors [20]).

##### Recovery of pre-fracture status

**Fig. 1:**
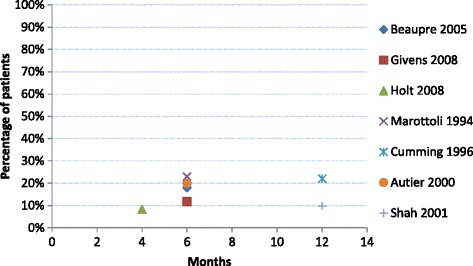
Percentage of surviving patients newly residing in nursing homes in the period following hip fracture, as reported in cohort studies. NB. Shah 2001 is for ambulatory, community dwelling participants pre-fracture; Holt 2008 represents 2 cohorts aged 75–89 and ≥95 years

In many industrialised Western nations (Canada, USA, Scotland, Australia, Belgium, Netherlands), approximately 10 to 20 % of people with hip fracture are newly institutionalised by six to 12 months following hip fracture (Fig. 1) [15, 18, 20, 25, 47, 50, 52]. Three studies reported accommodation outcomes for hip fracture patients at multiple time points post fracture (Additional file 1: Table S1). A small Swedish study reported approximately 80 % of survivors remained living in their own homes 4 months after hip fracture, and this proportion remained relatively constant for 10 years [31]. A study conducted in Japan found the proportion of patients living in residential care was similar before and after fracture (5 %, 7 % and 7 % living in residential care before, 1 year and 2 years post-fracture) [34]. This trend extended out to 10 years post-fracture [35].

#### Quality of life

##### Comparison to a non-fracture group

Two separate studies have found significant lower quality of life of people who have experienced hip fracture in comparison to control participants, over the longer term (Table 3) [19, 23].

##### Recovery of pre-fracture function

**Fig. 2:**
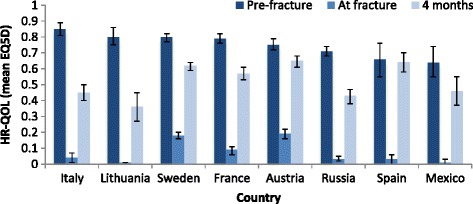
Health-related quality of life (HR-QOL) as measured by EQ5D before, after and at 4 months following hip fracture. *Source:* Borgstrom et al. [33]. NB. pre-fracture QOL was determined retrospectively

An international study (the International Costs and Utilities Related to Osteoporotic Fractures Study, ICUROS) demonstrated that quality of life in people 50 years or over sustaining a fracture from low energy trauma decreased significantly after 4 months in all of the eight countries (Fig. 2) [33]. The cumulative quality of life (EQ5D) loss ranged from 0.11 in Austria and Spain (95 % CI 0.10–0.12 and 0.07–0.14, respectively) to 0.21 (95 % CI 0.19–0.22) in Lithuania and 0.20 in Italy (95 % CI 0.19–0.21). Data from the UK and Sweden indicated a quality of life loss of 0.22 over the first year post-fracture (mean difference EQ5D −0.22, 95 % CI 0.17–0.26, UK data [46]; 0.22, 95 % CI 0.20–0.25, average annual loss over first 12 months post-fracture, Swedish data [36]).

## Discussion

The current review provides clear evidence that people recovering from hip fracture experience ongoing limitations in mobility, basic activities of daily living, self-care, participation and quality of life. Between 40 and 60 % of hip fracture survivors are likely to recover their pre-fracture level of mobility [25, 37, 47]. Up to 70 % of people may regain their pre-fracture level of independence for composite measures of basic ADL [22, 48, 51], but this proportion is likely to be lower for those with higher levels of dependence pre-fracture [48]. Half or fewer people experiencing hip fracture may regain their pre-fracture level of independence in IADLs as determined by composite measures [14, 38, 47]. For those highly independent pre-fracture, more than 70 % may recover their self-care independence for particular ADL activities (eg. putting on pants, cooking) [43]. Most people who recover their ability to perform basic or instrumental ADLs do so within the first 6 months after discharge, although the time to recovery for individual ADLs ranges from approximately 4 to 11 months [42, 43]. The limited data available indicate that participation in domestic life is greatly decreased after hip fracture and that it remains at a relatively constant level, in terms of proportion of survivors, from 4 months post-fracture [31, 43]. Few studies were identified that provided information on participation in community, social or civic life over the longer term for people experiencing hip fracture. Studies in many countries world-wide have indicated that hip fracture has a significant impact on quality of life in the medium- to longer-term. The impact of hip fracture on accommodation is likely to be dependent upon cultural factors. In Western nations, between 10 and 20 % of hip fracture patients are institutionalised within 6 to 12 months post-fracture.

Most identified studies were of community-dwelling individuals or mixed cohorts with a minority of people from residential care settings. Studies that directly compared those living in nursing homes with those from the community have reported much lower recovery rates for mobility, basic activities of daily living and self-care for those from residential care [48, 49]. A large US series of people from residential care, including 12 % who did not receive surgical intervention, reported that only 21 % of people living in residential care both survive and recover their mobility [28]. Similarly, less than 20 % survive and recover their independence for various activities of daily living. Differences in outcomes may be due to different levels of pre-fracture function or to different intervention approaches and intensities. However, it is difficult to directly compare the data from this study to those reported in other cohorts as the proportions are not determined as the percentage of survivors as reported in the other studies and the cohort includes data from those who did not receive surgical intervention [28].

Quantifying the degree of disability in this review over the medium to long-term was difficult due to the wide variations between the identified studies. Many patient and treatment level characteristics are expected to vary between studies, due to different inclusion criteria for the cohorts, including different age criteria, community or mixed residential settings pre-fracture and whether or not a series only includes those receiving surgical management. However, interpretation was further hampered by inconsistent methods of measuring and reporting outcomes. For example, a variety of definitions and methods of measurement for mobility were used, including walking different distances, walking with or without aids, walking indoors or outdoors and various combinations of these (see Additional file 1: Tables S1 and S3). Outcomes were also reported as change from pre-fracture function as either an improvement or decline or as absolute rates of independence or disability for different functions at various follow-up times, or even by the mean scores on the scales used. Future studies should attempt to measure the proportion of patients that regain their pre-fracture level of function or participation to enable comparisons of outcome rates across different study populations and settings. For example, two separate studies conducted in Canada and Australia reported outcomes for hip fracture patients admitted from long term care in comparison to those from the community [48, 49]. The estimate of the proportion walking independently without an aid pre-fracture varied widely between these studies, being 61 % for those from the community in the Canadian study and 96 % in the Australian study. Despite the large differences in function between the study populations at baseline, the proportion recovering is quite similar; the Canadian study reported that 71 % of those in the community recovered their level of pre-fracture function as measured by the MBI and the Australian study found 69 % recovered their prior level of ambulation. In addition, some studies did not clearly report the loss to follow-up of participants for each outcome; adherence to STROBE reporting guidelines would enable more accurate estimates of the range of likely recovery rates to better inform patients and clinicians alike.

Whilst previous reviews have summarised the impact of particular interventions to improve outcomes, [53, 54] or summarised particular outcomes for hip fracture patients, [54, 55] to the authors knowledge this is the first review to attempt to comprehensively summarise the medium to long term disability outcomes for hip fracture patients. This summary will enable clinicians and policy makers to gain an overview of likely outcomes and impacts of hip fracture for patients. In addition, summarising the outcomes for hip fracture according to the current WHO ICF Framework enables readers to understand outcomes from the perspectives of both the person and the person in society. The current review is not without limitations. This review was not conducted systematically and therefore cannot be assumed to be a completely comprehensive review of all studies. However, it provides a comprehensive overview of outcomes according to the ICF framework and includes the key studies reporting extensive information on functional recovery following hip fracture. The authors are unaware of any other reviews providing a similar summary of long-term disability outcomes after hip fracture. No studies were identified from Africa and only a few included from South East Asia or South America. We did not attempt to analyse findings by gender, although most studies included more than three quarters women and it is known that mortality rates are higher for men than women [45, 56, 57]. Pooling of study outcomes was not performed due to the wide variation in the type and reporting of outcomes used. This review did not examine predictors or risk factors for recovery or ongoing loss of mobility, function, life participation or changes in accommodation.

Most of the studies that report on disability outcomes are limited to those who survive to the specified outcome time point. Thus, this report is on the disabling effects of hip fracture on those who survive. However, it is important to consider that as many as 47 % may have died prior to 1 year, depending on the population being studied [28, 35, 58, 59]. Mortality rates for the included studies for the first 12 months post-fracture were generally approximately 10–20 %. Mortality in a series of patients from Brazil was reported as 35 %; in this series the median time between fracture to surgery was 9.5 days (interquartile range 6 to 17 days) [39]. A study of more than 60, 000 people experiencing hip fracture in residential care reported mortality rates of 36 % at a median of 4 months and 47 % died within 1 year [28]. In addition, it is likely that the least healthy patients are those that are lost to follow-up, therefore the study estimates may contain bias by including data from healthier survivors and are likely to provide an upper estimate of the recovery rates following hip fracture [43].

This report was limited to observational studies that were conducted under the usual care conditions of the study sites. It does not attempt to examine the relative effectiveness of different approaches to care. As the rehabilitation interventions received by study participants are varied these studies tell us about the range of current outcomes rather than potential for improvement with different approaches to intervention. Previous reviews have reported on interventions designed to improve outcomes following hip fracture [54, 60]. While there is much data available on the long-term recovery of mobility and basic ADLs for people experiencing hip fracture, data on the long-term impact on IADLs and life role participation are scarce. There is a need for further research on the consequences of hip fracture to inform policy makers and planners preparing for the increasing numbers of older people with disability resulting from hip fracture likely to be part of our society in the future. There is also a need for consistency in outcome measures to enable comparison and pooling of studies and outcomes across and between regions.

## Conclusions

This review highlights that while a proportion of people recover their pre-fracture function following hip fracture, for many the outcome from hip fracture is relatively poor. Future studies should determine the proportion of people that regain their pre-fracture level of functioning or participation to enable comparisons of outcomes between study populations and settings. There is a need to invest in research into interventions and programs designed to improve the longer term functional recovery of people following hip fracture, particularly given the increasing impact this is likely to have on our societies as the population demographics change.

## Additional file

Additional file 1:  Table S1. Outcomes from studies reporting activity, participation and accommodation outcomes at multiple follow-up times after hip fracture. **Table S2.** Medium to long-term functional outcomes for hip fracture patients in comparison to pre-fracture baseline, from prospective population-based cohort studies. **Table S3.** Medium to long-term function outcomes for hip fracture patients from studies reporting outcomes for those living in residential care (percentage of survivors). **Table S4.** Additional data on time to recovery of function following hip fracture as estimated from cohort studies. **Table S5.** Participation outcomes at single time-points post-fracture for countries or outcomes otherwise poorly represented. (PDF 348 kb)

## Abbreviations

ADL: Activities of daily living
HR: Hazard ratio
IADL: Instrumental activities of daily living
ICF: International classification of functioning, disability and health
OR: Odds ratio
QALY: Quality adjusted life year
QOL: Quality of life
US: United States
WHO: World Health Organization
