# Supplementary material for: A critical review of the long-term disability outcomes following hip fracture
Source: BMC Geriatr. 2016 Sep 2;16(1):158. doi: 10.1186/s12877-016-0332-0 (PMC5010762; doi:10.1186/s12877-016-0332-0)
Supplement: Additional file 1: Table S1. — Outcomes from studies reporting activity, participation and accommodation outcomes at multiple follow-up times after hip fracture. Table S2. Medium to long-term functional outcomes for hip fracture patients in comparison to pre-fracture baseline, from prospective population-based cohort studies. Table S3. Medium to long-term function outcomes for hip fracture patients from studies reporting outcomes for those living in residential care (percentage of survivors). Table S4. Additional data on time to recovery of function following hip fracture as estimated from cohort studies. Table S5. Participation outcomes at single time-points post-fracture for countries or outcomes otherwise poorly represented. (PDF 348 kb) [file 12877_2016_332_MOESM1_ESM.pdf]

Supplementary Table 1 Outcomes from studies reporting activity, participation and accommodation outcomes at multiple follow-up times after hip fracture

Study	Outcome	Pre-fracture	Follow-up time					
			3-5 mo	6-9 mo	9-18 mo	19 mo – 2 yrs	3-5 years	6-10 years
Activity - Mobility								
Borgquist 1990	Walking (% survivors)	95%	73%		80%		80%	76%
Griffin 2015	Walking regularly:							
	indoors without aids ≤80 years	66%	37%		49%			
	indoors without aids >80 years	46%	14%		20%			
	outdoors without aids ≤80 years	51%	18%		26%			
	outdoors without aids >80 years	29%	6%		8%			
Kitamura 1998	Walking alone outdoors (± assistive device)	68%	51%		58%	58%		
	Walk alone outdoors + helper	8%	13%		10%	8%		
	Walk alone indoors (± assistive device)	11%	10%		7%	9%		
	Walk alone indoors + helper	8%	10%		8%	6%		
	Sit/wheelchair	3%	11%		9%	11%		
	Bedridden	2%	5%		8%	8%		
Tsuboi 1998	Walking alone outdoors (± assistive device)	68%	51%		56%			
Magaziner 1990	Walk independently or with 1 stick	87%	26%[2]		54%			
Magaziner 2003	Walking 3m without assistance	74%			46%	47%		
Neuman	New total locomotion dependence		28%		27%			
Samuelsson 2009	Walking independent/1 stick	51%	25%			28%		
	2 sticks/frame	45%	61%			54%		
	Non-walking	4%	15%			18%		

Study	Outcome	Pre-fracture	Follow-up time					
			3-5 mo	6-9 mo	9-18 mo	19 mo – 2 yrs	3-5 years	6-10 years
Activity - Composite measures of Basic ADLs								
Doshi 2014	Mean MBI (those surgically treated)	91.2%		80.8%	87.0%			
Koval 1998	Mean functional recovery score (composite measure of mobility 33%, BADLs 44% and IADLs 23%)<br>Mean functional recovery (functional recovery score as % of pre-fracture score)	83.8 (range 8.25-100)	77%	86%	86%			
Magaziner 1990	Fully independent in Basic ADLs	70%	14%[2]		40%			
Norton 2000	Independent (% survivors)	62%				44%		
Miller 2009	Lower extremity ADLs (no. of tasks requiring assistance) (SD)							
	No falls	4.4 (3.4)		7.0 (3.3)	6.9 (3.4)			
	1 fall	4.8 (3.4)		8.0 (2.7)	6.7 (2.9)			
	>1 fall	5.6 (3.2)		8.7 (3.0)	9.2 (2.9)			
Samuelsson 2009	ADL-independent	74%		58%		59%		
Activity - Self-care								
Borgquist 1990	Dressing/personal hygiene	89%	83%		88%		83%	92%
Magaziner 2003	Requiring grooming assistance (SE)	78% (1%)			17% (2%)	18% (2%)		
Participation – Composite measures of IADLs								
Magaziner 1990	Fully independent in IADLs	34%	4%[2]		14%			
Participation – domestic life								
Borgquist 1990	Cooking/washing dishes	74%	63%		60%		62%	70%
	Cleaning/laundry	59%	47%		48%		53%	54%
	Shopping	67%	39%		53%		52%	68%
	Need for social services	-	33%		32%		23%	35%

Study	Outcome	Pre-fracture	Follow-up time					
			3-5 mo	6-9 mo	9-18 mo	19 mo – 2 yrs	3-5 years	6-10 years
Participation – community, social and civic life								
Borgquist 1990	Visiting someone monthly (those admitted form own home)	74%					67%	76%
Miller 2009	No. social activities over 2 weeks (SD)							
	Overall			9.2 (10.0)	8.9 (SD)			
	Those with no falls over 6 months	10.5 (8.7)		10.2 (10.0)	10.1 (10.2)			
	1 fall over 6 months	10.9 (12.6)		8.9 (11.1)	8.5 (10.9)			
	≥2 falls over 6 months	6.5 (5.3)		3.5 (5.7)	3.9 (5.0)			
Accommodation								
Borgquist 1990	Own home (% total)		79%		76%		43%	30%
	Old peoples home		6%		5%		9%	4%
	Nursing home		5%		8%		5%	2%
	Hospital		7%		0%		0%	0%
Kitamura 1998	Own home	84%	76%		86%	85%		
	Hospital	11%	18%		7%	8%		
	Residential care	5%	6%		7%	7%		
Tsuboi 2007	Own home	84%	60%		81%			86%
Samuelsson 2009	Own home							
	Women	77%	79%		63%			
	Men	82%	86%		75%			

[1] Data also reported by age groups for walking regularly with one aid, with two aids or frame and not walking
[2]Two months post-discharge
Abbreviations: ADL = activities of daily living, BOAS = Brazil Old Age Schedule, LTC = long term care, MBI = modified Barthel Index, mo = months, TICS = Telephone Interview to Assess Cognitive Status, yrs = years

Supplementary Table 2 Medium to long-term functional outcomes for hip fracture patients in comparison to pre-fracture baseline, from prospective population-based cohort studies

Study	Outcome	Pre-fracture	Follow-up time	
			3-6 mo	1 – 2.5 yrs
Activity - Mobility				
Bentler 2009	Mean no. abilities with difficulty	1.70		2.63
Marottoli 1992	Walking across room independently	75%	15%	
	Walk across room ± mechanical assistance	96%	74%	
	transfer independently	90%	32%	
	transfer ± mechanical assistance	96%	68%	
	Climb flight of stairs independently	63%	8%	
	Walk half mile independently	41%	6%	
Wolinsky 1997	Mean increase in no. limitations:			
	Lower body			1.75
	Upper body			0.50
	Mean adjusted increase in no. limitations due to hip fracture:			
	Lower body			0.93
	Upper body			0.26
Bentler 2009	Mean no. of functional abilities with difficulty	0.67		1.54
	Mean increase in no ADLs with difficulty			0.88
Wolinsky 1997	Mean increase in no. ADL limitations			2.08
	Mean adjusted increase in no. limitations due to hip fracture			1.12
Activity - Self-care				
Marottoli 1994	Dressing independently	86%	49%	
	Dressing ± mechanical assistance	86%	51%	
	For those who could perform 4 or 5 items independently at baseline:			
	Dressing independently	99%	60%	
	Dressing ± mechanical assistance	99%	62%	
Participation – domestic life				
Wolinsky 1997	Mean increase in no. household ADL limitations			0.89
	Mean adjusted increase in no. household ADL limitations due to hip fracture			0.35

Study	Outcome	Pre-fracture	Follow-up time	
			3-6 mo	1 – 2.5 yrs
Participation – Composite measures of IADLS				
Bentler 2009	Mean no. functional abilities with difficulty	0.65		1.32
Wolinsky 1997	Mean increase in no. advanced ADL limitations<br>Mean adjusted increase in no. advanced limitations due to hip fracture			0.44<br>0.21
Health condition				
Bentler 2009	Mean self-reported health status<br>Mean cognition (TICS/15)	60.6<br>12.9		56.1<br>12.6
**Accommodation**				
Marottoli 1994	Insitutionalised during 6 months post-fracture (% total)<br>(% survivors)		29%<br>23%	

Supplementary Table 3 Medium to long-term function outcomes for hip fracture patients from studies reporting outcomes for those living in residential care (percentage of survivors)

Study	Outcome	Pre-fracture residence	Pre-fracture	4 months	6 months	1 year
Activity - Mobility						
Crotty 2000	Independent ambulation	Residential care	61%	32%		
		Home	96%	70%		
	Assistance for ambulation	Residential care	34%	32%		
		Home	4%	24%		
	Unable to ambulate	Residential care	5%	36%		
		Home	0%	6%		
Beaupre 2007	Walking without assistive devices	Residential care	39%		4%	
		Community	61%		26%	
	Walking ± assistive devices	Residential care	79%		45%	
		Community	97%		89%	
	Chair transfers	Residential care	80%		47%	
		Community	97%		92%	
	Toilet transfers	Residential care	44%		15%	
		Community	96%		88%	
	Stair climbing	Residential care	56%		15%	
		Community	92%		77%	
Neuman 2014	New total locomotion dependence	Nursing home		27.8%		26.5%
Activity - Composite measures of Basic ADLs						
Beaupre 2007	Adjusted relative reduction in pre-fracture function (95%CI)	Residential care			33% (27-41)	
		Community			12% (8-15)	
Crotty 2000	Independent in all BADLs	Residential care	3%	2%		
		Home	46%	28%		
Participation – self-care						
Beaupre 2007	Dressing	Residential care	35%		9%	
		Community	88%		73%	
	Hygiene	Residential care	51%		22%	
		Community	94%		90%	
	Bathing	Residential care	9%		1%	
		Community	71%		48%	
	Feeding	Residential care	59%		43%	

		Community	94%		89%	
	Managing bowel continence	Residential care	52%		25%	
		Community	88%		85%	
	Managing bladder continence	Residential care	36%		15%	
		Community	74%		75%	
Accommodations						
Crotty 2000	Admitted to higher level care	Residential care		16%		
		Community		14%		

Supplementary Table 4 Additional data on time to recovery of function following hip fracture as estimated from cohort studies

Study	Outcome Measure	Function	Outcome
Magaziner 1990	Patients recovering or experiencing a decline in ability over the period 6-12 months post-discharge	**Activity- mobility**	
		recover previous walking ability	10.5%
		decline in walking ability	10.1%
		**Activity – composite measures of BADL**	
		improving ≥2 points in BADLs	12.9%
		declining ≥2 points in BADLs	14.3%
		**Participation – composite measure of IADL**	
		improving ≥2 points in IADLs	20.9%
		declining ≥2 points in IADLs	19.5%
Magaziner 2000	Recuperation time estimated using longitudinal analysis to estimate the point in time at which additional recovery not detected	**Activity- mobility**	
		chair rise speed	9.9 months
		walking speed	10.8 months
		walking 3m without assistance	13.1 months
		**Activity – composite measures of BADL**	
		upper extremity ADLs	4.3 months
		lower extremity ADLs	11.2 months
		**Health condition**	
		Depressive symptoms	3.9 months
		cognitive status	4.4 months

Supplementary Table 5 Participation outcomes at single time-points post-fracture for countries or outcomes otherwise poorly represented.

Study	Outcome	Pre-fracture	Follow-up time 1-2 yrs	2-3 yrs
Mobility				
Suriyawongpaisal 2003	Walking with support	1.2%	14%	
	Use wheelchair	1.2%	23.2%	
	Transfer assisted	0.8%	11.2%	
Self-care				
Suriyawongpaisal 2003	Bathing assisted	0.8%	11.2%	
	Tooth-brushing assisted	0	4%	
	Dressing assisted	1.2%	10%	
	Feeding assisted	0	4.8%	
	Toileting assisted	0.8%	21.6%	
	Fecal incontinence	0	3.2%	
	Urinary incontinence	1.6%	6.8%	
Wang 2015	Daily life self-care (% survivors)			
	Independent			59.4%
	Partially disabled			36.0%
	Disabled			4.6%
Participation – domestic life				
Keene 1993	Retain unaided mobility		40%	
	Shopping (% survivors)	54%	33%	
	Return to own home (%total)		43.5%	
	Residential care	21.4%	19.9%	
